# Aggressive Behavior of Recurrent Middle Cerebral Artery Aneurysms: A Case Series

**DOI:** 10.7759/cureus.76435

**Published:** 2024-12-26

**Authors:** Alejandro Serrano-Rubio, Héctor A Rodríguez-Rubio, Rodrigo López-Rodríguez, Juan Carlos Balcázar Padrón, Sharon Trujillo, Ana M Martínez-Cáceres, Brenda Susana Hernández-Barrera, Rafael Sánchez-Mata, Daniel Figueroa-Zelaya, Edgar Nathal

**Affiliations:** 1 Vascular Neurosurgery, National Institute of Neurology and Neurosurgery, Mexico City, MEX; 2 Neurological Surgery, National Institute of Neurology and Neurosurgery, Mexico City, MEX

**Keywords:** aneurysm bifurcation, aneurysm recurrence, aneurysm remnant, middle cerebral artery, rebleeding

## Abstract

The bifurcation of the middle cerebral artery (MCA) is one of the most common sites for the formation of cerebral aneurysms, presenting significant challenges for surgical management. A primary concern in this area is the recurrence of aneurysms following surgical clipping, which necessitates a thorough investigation of the contributing factors. This study examined all cases of rebleeding from previously clipped MCA aneurysms among 195 surgically treated patients over an 11-year period. Our evaluation utilized detailed clinical records, imaging studies, and surgical video documentation. We specifically analyzed the direction of the aneurysmal dome, the type of clip used, clipping angles, and various factors that may contribute to rebleeding. Out of the 195 cases, three patients (1.5%) experienced rebleeding within four to 12 months following the initial clipping. Among these three cases, one was evaluated using digital subtraction angiography (DSA) post-clipping, while the other two were assessed with computed tomography angiography (CTA). The recurrence cases were characterized by atypical and aggressive patterns, involving rapid morphological changes that necessitated additional interventions. Surgical management for these cases included re-clipping or aneurysmectomy combined with revascularization procedures to address the recurring aneurysms. The findings of this study highlight the importance of understanding the relationship between anatomical and technical factors in the surgical management of MCA aneurysms. By identifying patterns associated with recurrence, this research seeks to improve decision-making processes and optimize strategies for managing recurrent aneurysms after clipping. Differences in imaging techniques may have affected the sensitivity in detecting small residual necks. This comprehensive approach can potentially enhance long-term outcomes for patients receiving treatment for MCA aneurysms.

## Introduction

The middle cerebral artery (MCA) is a common site of aneurysms, representing 18% to 40% of all aneurysms; among these, MCA bifurcation is the most frequent location [1]. Surgical management of cerebral aneurysms offers an enduring result after clipping; however, recurrence has been reported after an apparent correct exclusion. The literature estimate of aneurysmal annual regrowth after a successful clipping is 0.26%-0.53%; this may occur because the clipping was incomplete and there’s residual neck or the wall is weak, while the annual risk of new aneurysm formation is 1%-2% [2,3]. Subarachnoid hemorrhage (SAH) resulting from aneurysm rupture is a catastrophic event that causes morbidity and sequelae and is associated with vasospasm, which could trigger the patient's death. However, the presence of SAH after the complete clipping of an aneurysm is rare, with an incidence of 2% within the first decade, and the incidence increases to 9%-12.4% in the 20th year [4,2]. Risk factors for remnants after clipping are well-known, but the causes of early and aggressive recurrence are unclear and represent a matter of concern [5]. This article presents a series of three cases of recurrence after microsurgical clipping of MCA aneurysms with complete occlusion, experiencing a new episode of SAH, requiring retreatment; our main objective is to report this unusual aggressive behavior of these aneurysms and to identify the risk factors involved in their recurrences.

## Case presentation

Materials and methods

We identified three cases (1.5%) of early recurrence and rebleeding among 195 MCA aneurysms treated over a 12-year period. These cases exhibited aggressive behavior, with rebleeding occurring between four and 12 months following the initial surgical clipping. All cases included comprehensive clinical records, imaging studies, and surgical video recordings, which provided a strong dataset for analysis. Of the aneurysms evaluated, 85% underwent post-clipping catheter digital subtraction angiography (DSA), while 15% were assessed using computed tomography angiography (CTA). We examined factors such as dome direction, clip type, clipping angles, and other anatomical and surgical elements related to recurrence to identify potential contributing factors.

**Figure 1:**
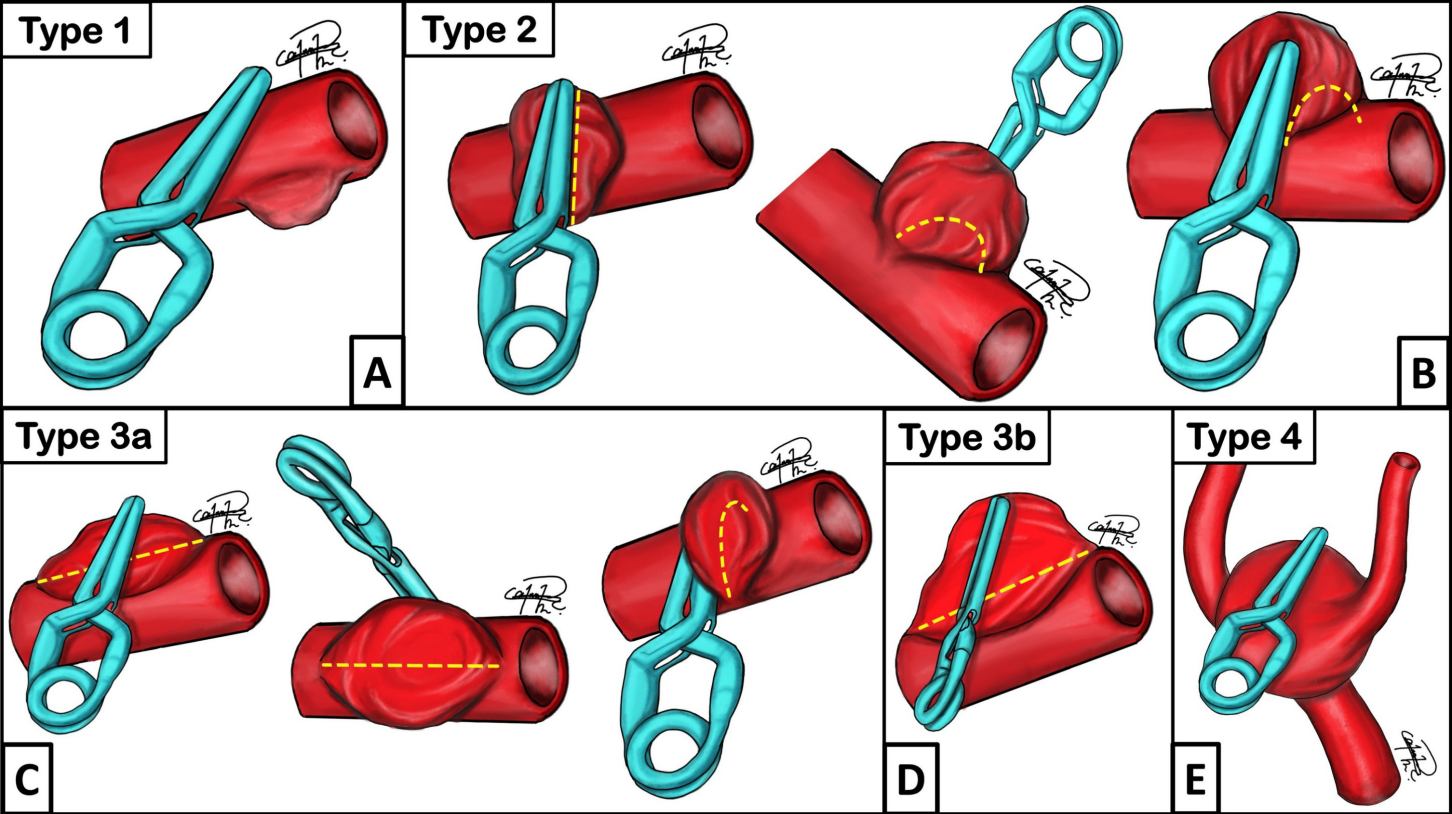
Classification system for recurrent aneurysms Classification system by Kobayashi et al. [6]. The closure line is defined as the line formed on the aneurysm by clipping, and the closure plane is the circumference of the recurrent aneurysm neck (yellow dotted line). (A) Type 1: The recurrent aneurysm is located in a different area than where the clip was initially placed or in contact at only one point. (B) Type 2: The closure line and the closure plane are almost identical. (C) Type 3a: The closure line intersects with the closure plane. (D) Type 3b: In rare cases, the current closure line is far enough away from the entire circumference of the closure plane. (E) Type 4: There is no reconstructive closure line available. The original illustrations were created by Héctor A. Rodríguez-Rubio

The classification systems developed by Kobayashi (Figure 1) and Spiotta were used to categorize the patterns of recurrence. These classifications were applied retrospectively for this study [6,7]. Although retrospective classification limits its predictive application for initial treatments, it offered valuable insights into recurrence mechanisms and helped inform subsequent reconstructions. These classification models were selected for their capacity to provide detailed characterization of aneurysm remnants and mechanisms of regrowth, thereby facilitating an understanding of the failure modes of initial surgical treatment. The Spiotta classification categorizes aneurysms based on the localization of residual remnants relative to the clip [7]. Type 1 describes proximal remnants caused by suboptimal clip placement, which may result from incomplete visualization or misjudgment during the initial procedure. Type 2 involves remnants distal to the clip blades, often attributed to insufficient sealing of the aneurysm dome. Type 3 refers to lateral remnants adjacent to the primary clipping line, reflecting incomplete occlusion or misalignment of the clip. Conversely, the Kobayashi (Figure 1) classification provides a broader perspective by categorizing recurrent aneurysms based on their growth patterns relative to the clip's closure line and the original aneurysm anatomy [6]. Type 1 (Figure 1A) involves recurrence at a different location from the initial clipping site, which may represent de novo aneurysm formation or hemodynamic alterations in adjacent vascular segments. Type 2 (Figure 1B) recurrence occurs beneath the clip blades, either proximally, distally, or along the entire blade closure line, implicating inadequate occlusion pressure or potential clip migration. Type 3a (Figure 1C) is characterized by lateral growth of the remnant opposite the closure line, whereas type 3b (Figure 1D) refers to remnant growth that causes separation between the clip and the original aneurysm circumference. Type 4 (Figure 1E) recurrence is characterized by diffuse regrowth in all directions, leading to the development of a new aneurysmal structure, which may be due to ongoing hemodynamic stress or inherent fragility of the vascular wall.

Results

Case 1

**Figure 2:**
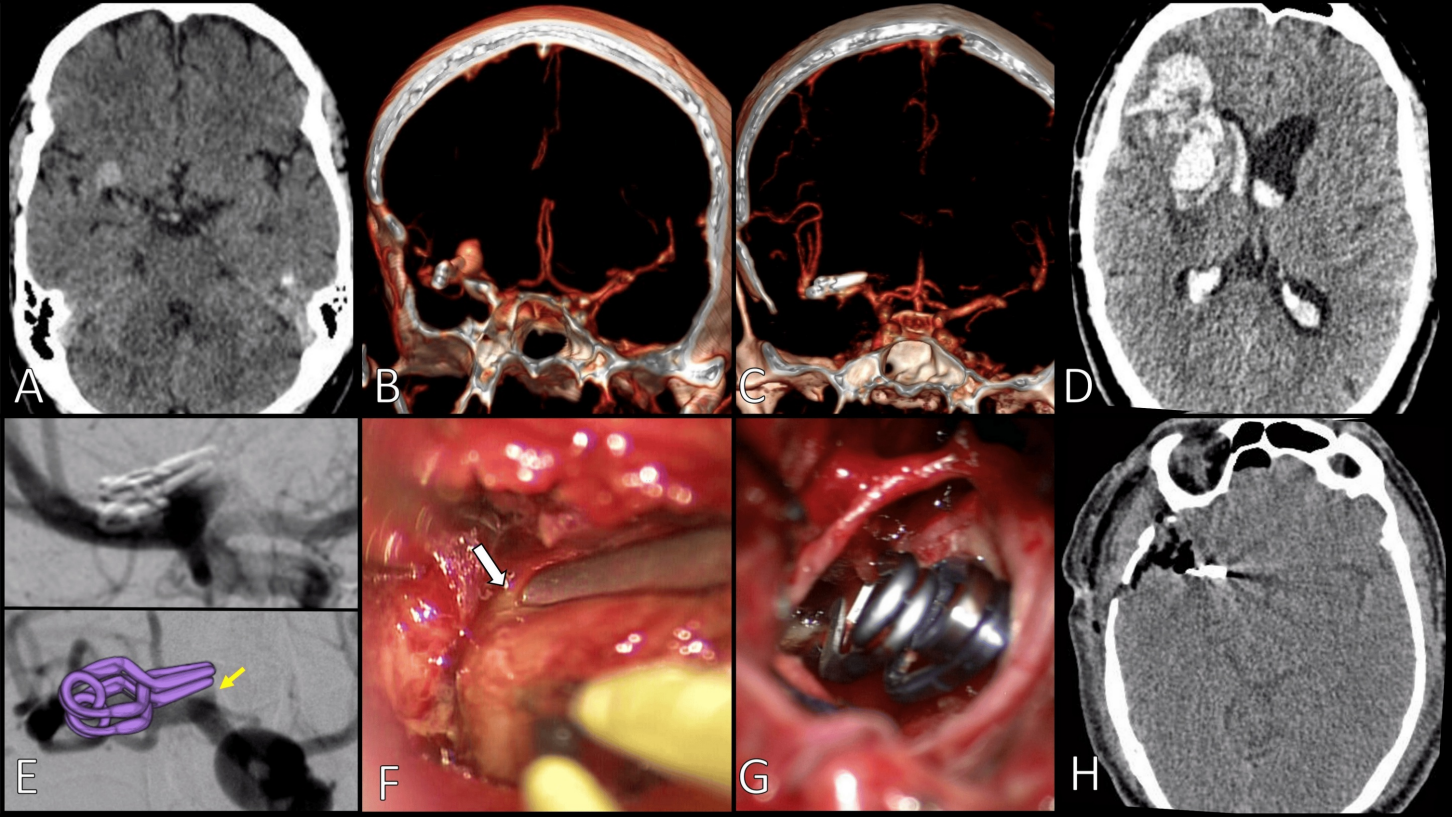
Case 1. (A) CTA showing a small hematoma in the right MCA bifurcation territory. (B) A 3D reconstruction showing an old clip dating from 2004 at the right MCA with a recurrent aneurysm. (C) No evidence of remnant aneurysm. (D) CT scan with a new SAH Fisher 4. (E) DSA images, (up) clip position enhancement (low), show a small residual at the base of the neck (arrow). (F) A remnant aneurysm was observed ahead of the clip tip (arrow). (G) Final view after re-clipping. (H) Postoperative CT scan MCA: middle cerebral artery; CTA: computed tomography angiography; DSA: digital subtraction angiography; 3D: three dimensional; SAH: subarachnoid hemorrhage; CT: computed tomography

A 58-year-old male with hypertension, diabetes, and a history of smoking underwent clipping of the right MCA aneurysm 16 years prior at an external institution. Two days before admission, the patient experienced a thunderclap headache with associated nausea and vomiting (Figure 2). CTA revealed a Fisher grade 2 subarachnoid hemorrhage (SAH) and a saccular aneurysm of the right MCA (Figures 2A-2B). The aneurysm was treated with two 7 mm Yasargil clips, with a 3D reconstruction of the CTA that demonstrated no evidence of a residual neck (Figure 2C). However, DSA was not performed to confirm the complete exclusion of the aneurysmal neck, which may have limited the detection of smaller remnants. Four weeks later, the patient returned with a holocranial headache, vomiting, and tetraparesis. Imaging demonstrated a Fisher grade 4 SAH with recurrent aneurysm formation at the previous clipping site (Figures 2D-2E). Intraoperatively, a residual neck was identified, alongside a fragile aneurysmal wall, consistent with Kobayashi type 2 (Figure 1B) and Spiotta type II (distal) classifications (Figures 2F-2G). The recurrent aneurysm was clipped, and the hematoma was evacuated. Postoperatively, imaging revealed a low-density area at the hematoma site, and the patient achieved a modified Rankin Scale (mRS) score of 2, indicating slight disability with functional independence (Figure 2H). Follow-up imaging included two DSA and one CTA. The first DSA, one-month postsurgery, confirmed complete aneurysm occlusion and vascular integrity. A second DSA at 12 months and a CTA at 24 months showed no evidence of recurrence or new aneurysmal formation. The patient remains clinically stable under long-term surveillance.

Case 2

**Figure 3:**
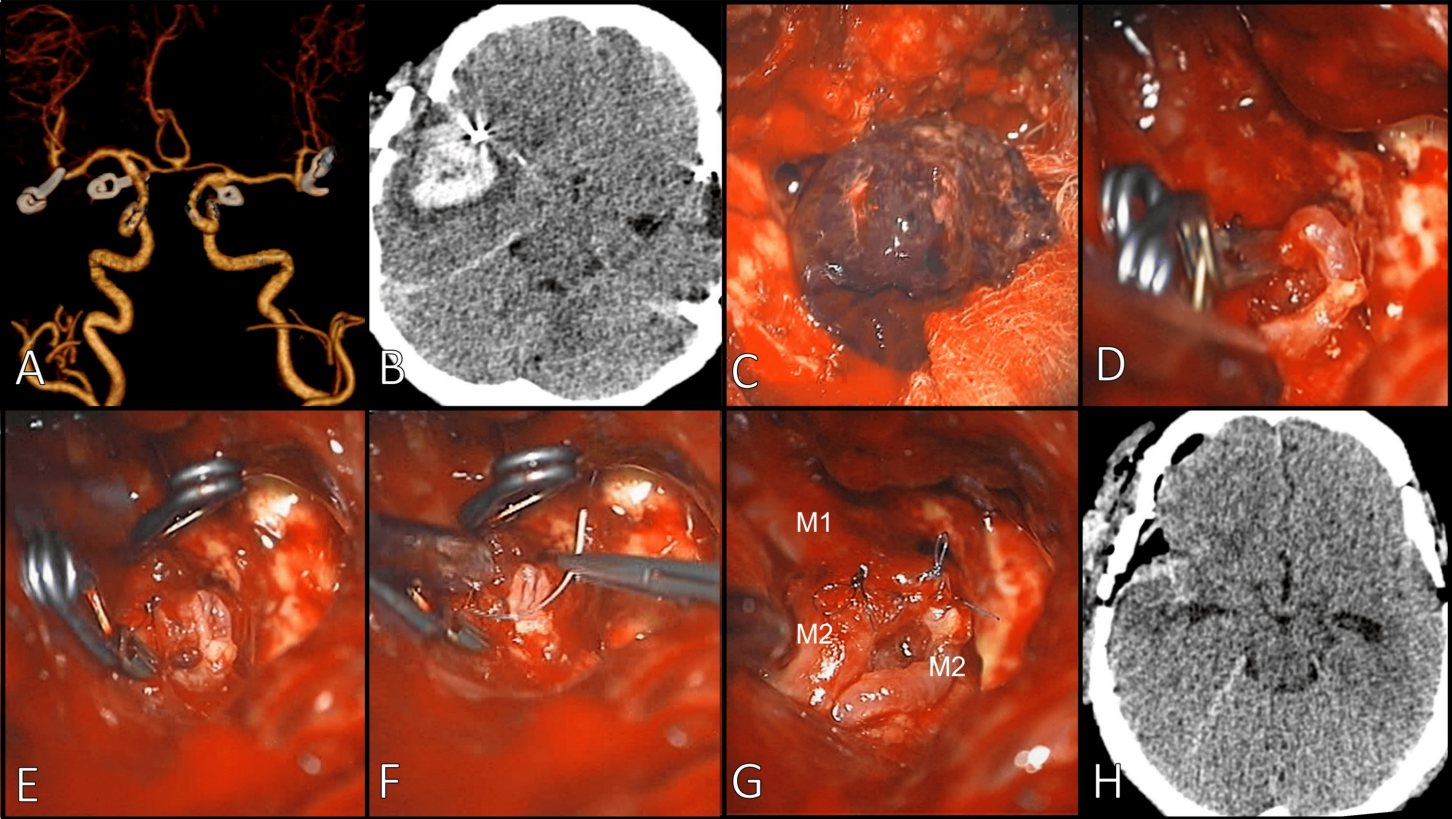
Case 2. (A) Brain CTA during follow-up showing a complete clipping in a case with multiple aneurysms at MCA and PCom bilateral location. (B) Six months later, a new SAH episode occurred in the right MCA territory. (C) Intraoperative view of the intracerebral hematoma around the aneurysm. (D) Isolation of M1-M2 segment. (E) Resection of the remnant aneurysm. (F) Preparation for an end-to-end anastomosis. (G) Complete anastomosis showing the M1 and both M2 segments of the MCA. (H) Postoperative brain CT scan MCA: middle cerebral artery; CTA: computed tomography angiography; PCom: posterior communicating artery; SAH: subarachnoid hemorrhage

A 36-year-old female with no significant medical history presented with multiple aneurysms, including a ruptured lesion in the right MCA (Figure 3). All aneurysms were sequentially clipped, and postoperative CTA confirmed complete occlusion with no residuals (Figure 3A). DSA was not performed, limiting the detection of any small remnants. Six months later, she presented with Fisher grade 4 SAH and a hematoma in the right Sylvian fissure (Figure 3B). During surgery, we evacuated the hematoma (Figure 3C), which was classified as Kobayashi type 2 (Figure 1B) and Spiotta type I (proximal). Due to a wide neck and atheromatous wall, the affected segment of the MCA was resected, and a successful end-to-end anastomosis was performed (Figures 3D-3G). One year later, the patient developed SAH from a de novo aneurysm in the distal anterior cerebral artery, which was clipped (Figure 3H). Postoperative imaging was limited to CTA; the patient remains under follow-up without further complications.

Case 3

**Figure 4:**
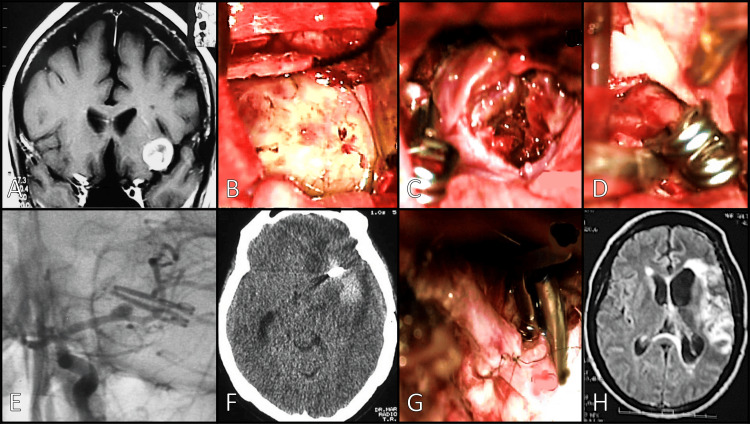
Case 3. (A) MRI coronal view showing a giant saccular aneurysm in left MCA. (B) Intraoperative exposure of the dome. (C) Aneurysmectomy and evacuation of the thrombus. (D) Two straight clips and a residual aneurysm were observed. (E) Postoperative DSA showing two straight clips and residual aneurysm. (F) Brain CT showed a new SAH episode with a surrounding hematoma in the left MCA. (G) MI-M2 end-to-end anastomosis. (H) Postoperative brain MRI MRI: magnetic resonance imaging; SAH: subarachnoid hemorrhage; DSA: digital subtraction angiography; CT: computed tomography; MCA: middle cerebral artery

A 43-year-old female patient presented to the emergency room due to an explosive holocranial headache associated with nausea, vomiting, and neck stiffness (Figure 4). An MRI showed SAH and a giant saccular aneurysm of the left M1 segment (Figure 4A). We approached the aneurysm through a standard pterional route. After opening the Sylvian fissure, the aneurysm was exposed (Figure 4B), and under temporary clipping, we completed an aneurysmectomy to resect a partially well-organized thrombus (Figure 4C). Finally, we clipped the aneurysm using two straight 11 mm clips (Figure 4D). A small residual neck was left intentionally due to a thick atherosclerotic wall that prevented the total occlusion because of a flow interruption in the parent artery detected through the intraoperative Doppler. The patient was discharged without complications. Four months later, the patient suffered another similar event: a thunderclap headache associated with nausea, vomiting, neck stiffness, right-side hemiplegia, and motor aphasia. Postoperative imaging studies showed a Fisher grade 4 SAH with intraparenchymal hemorrhage secondary to a rebleeding of the previously clipped left MCA aneurysm and regrowth of the aneurysm classified as Kobayashi type 3a (Figure 1C) and Spiotta type III (lateral) (Figure 4E). We treated the aneurysm for a second time, and four months later, a new episode of SAH was documented with a hematoma and a hypodensity area around it (Figure 4F). We considered a more radical solution by excluding the aneurysm through a complete resection with an end-to-end M1-M2 anastomosis (Figure 4G).

## Discussion

The main objective of treating intracranial aneurysms is to achieve complete occlusion and isolate them from cerebral circulation. Microsurgical clipping is considered the standard procedure for addressing these aneurysms. However, postoperative angiography has revealed that 4% to 8% of patients still have residual aneurysms after the surgery [8,9]. Some studies have reported that the rebleeding rate of aneurysms with residual necks was about 3.5%-28% [10] and that aneurysm regrowth occurred at 3.5%-15% [11]. Ebina et al. proposed that the fragility of the vascular wall, particularly at the clip edge subjected to hemodynamic stress, contributes to recurrent aneurysm growth. They highlighted that silver aneurysm clips could corrode and induce granulomatous tissue reactions, increasing recurrence rates. Modern aneurysm clips are now made from inert materials with high closing pressures, alleviating these concerns [12]. However, there can be slippage issues with titanium clips [13]. According to Feuerberg et al., an incomplete clipping rate varies from 1.6% to 14% [14]. Few data are available regarding the long-term DSA follow-up of successfully treated aneurysms with neck clipping. David et al. [15] reported an annual regrowth rate of 0.52% for completely clipped aneurysms in a series of 135 patients followed for 4.4 years. In another study with a mean follow-up of nine years, Tsutsumi et al. [9] reported an annual incidence of 0.26% in a series of 125 patients. In a recent case series involving nine recurrent intracranial aneurysms following successful neck clipping at a single center, the incidence was found to be approximately 0.02% per year. Aneurysms recurred after an average of 13.3 years [16].

Pathogenesis and classification

Intracranial post-clipping aneurysms are classified as either recurrent or residual. Recurrent aneurysms typically develop due to one of two primary factors: inadequate clipping that fails to sufficiently reinforce the parent artery and aneurysm neck, permitting subsequent regrowth, or structural compromise caused by the clip itself, which can lead to the formation of a new aneurysm [15,17]. Conversely, residual aneurysms arise from incomplete clipping or clip slippage, which is particularly common in cases involving wide or calcified aneurysm necks. In such scenarios, persistent blood flow can lead to the progressive expansion of the unoccluded portion of the aneurysm [17,18].

MCA aneurysms

MCA aneurysms account for 20% to 30% of all ruptured aneurysms and require different techniques to achieve adequate occlusion due to anatomic variants, different branching patterns, and hemodynamic abnormalities [18,19]. Among aneurysms arising from this artery, those at the bifurcation are the most common, accounting for 63% of the cases. The complete surgical obliteration is about 98.3%, where the remnants appear in 1.7% to 18%. Aneurysms with necks greater than 9 mm are associated with a higher residual MCA incidence [1]. Another concern about these aneurysms is their association with intracerebral hematomas when ruptured (present in almost 50% of the cases), with the consequent brain edema, making the surgical procedure more complicated. A total of 14% of them will require a decompressive craniectomy [20].

Regrowth rate and risk factors

Recurrent aneurysms develop at the original site despite postoperative imaging showing complete occlusion, whereas de novo aneurysms form in previously unaffected vessel segments [2]. The annual regrowth risk for fully clipped aneurysms is 0.26%-0.52%, compared to 0.89%-1.8% for de novo aneurysms [9,15]. Factors contributing to recurrence include aneurysm characteristics, clip material, surgical techniques, and imaging limitations. Residual necks, often undetectable on CTA due to their lower sensitivity and susceptibility to artifacts, are a key contributor to regrowth. Phynox clips exacerbate this limitation due to greater artifact production compared to titanium clips, underscoring the importance of angiography with rotational or multiangle imaging for accurate detection of subtle remnants. Kim et al. further identified aneurysm location as a significant risk factor, with 42.8% of recurrences occurring in the anterior communicating artery [21].

Some biological risk factors are associated with forming new aneurysms, such as smoking, hypertension, age under 45, and female gender [9]. Family history of aneurysm and multiplicity at the initial SAH were significant risk factors for de novo formation [22]. Tsutsumi et al. [9] noted that having multiple aneurysms at initial presentation and residual aneurysms after clip ligation are key risk factors for recurrence. On the other side, incomplete occlusion of the original aneurysm, a large size, and a wide neck are known risk factors for aneurysm recurrence [23,24]. Other factors preventing complete clipping include giant aneurysms and wide-thrombosed necks with branches that come out of the sac [4,7]. In these cases, the cause is merely mechanic because of an incomplete neck obliteration acting together with other factors such as the angle of the parent artery, vessel wall disease, blood pressure, and wall thickness.

Aggressive behavior 

We called aggressive behavior the accelerated regrowth associated with SAH in a short period (one year or less) after an apparent successful clipping. In most reports, the mean time between the first aneurysm and the appearance of a new aneurysm is reported to be up to 29 years after clipping, and the recurrence rate of SAH is 2.2% at 10 years and 9.0% at 20 years after the initial treatment [25,26]. However, in the present series, patients developed fast-growing aneurysms in a range of one to 12 months, and these were not diagnosed until the new hemorrhagic event occurred. These unexpected episodes of SAH could be related to previous aneurysmal wall remodeling, with wall deterioration because of smooth muscle cell apoptosis, extracellular matrix breakdown, and inflammatory cell growth [27]. Conversely, most intracranial dissections with SAH occur in the posterior circulation, possibly due to thinner media and adventitia in the intradural vertebral arteries compared to intracranial arteries in other locations. The histopathology of the vessel wall predicts the appearance of arterial dissections. On imaging, a typical dissection demonstrates irregularity of the luminal surface and a proximal segment of stenosis followed by a fusiform dilatation [28,29]. Remnants of aneurysms, such as dog-ear-shaped structures, generally remain stable and carry a bleeding risk of approximately 1.5% per year. In some cases, these remnants regress or disappear over time. The angle between the aneurysmal dome and the parent artery plays a crucial role in determining whether these remnants progress or resolve [1,30,20]. 

Classification schemes

Spiotta et al. [7] categorized 26 recurrent aneurysms from two institutions into three types: type I, proximal to the clip; type II, distal to the clip; and type III, lateral. In this work, we evaluated the type of recurrence using the classifications of Spiotta [7] and Kobayashi [6] (Figure 1), as described above. According to Spiotta, the most common type of recurrence was distal to the clip tip (46.1%), while the least frequent occurrence was proximal to the clip tip (19.2%). Type III (lateral regrowth) was found in (34.6%) of cases [27]. In this series, we had one case with Spiotta’s type I (case 2), one with Spiotta’s type II (case 1), and another one with Spiotta’s type III (case 3). According to Kobayashi's classification, we had two cases with Kobayashi’s type 2 (cases 1 and 2) (Figure 1B) and one with Kobayashi’s type 3a (case 3) (Figure 1C). The reconstruction was made in cases 1 and 2 with straight clips using the same closure plane. Case 3 represented Kobayashi’s type 3a, in which a tangential reconstruction was performed because the closure line intersected with the closure plane. El-Beltagy et al. [16] identified nine cases of recurrent aneurysms after initial clipping, classifying them into three types based on the relationship with the old clip: type A, the old clip occupies the new aneurysm neck.; type B, the old clip located far from the aneurysm neck; and type C, the location of the old clip, is independent of the recurrent aneurysm neck. They concluded that the old clip should be removed in type A, may not need removal in type B, and does not prevent new clipping in type C. 

Surgeon recommendations

Concerning the surgical technique, some authors suggest not removing the old clips [30]. Alternatively, we release the fibrous tissue covering the old clip with a scalpel before removal to avoid neck ruptures. In addition, the prior opening of the Sylvian fissure and basal cisterns, as in our case 2, could be challenging due to adherent fibrous tissue [15,31]. There are no established standards for evaluating and detecting remnant necks during follow-up. However, it is imperative to perform control imaging studies in the postoperative period and late follow-up. Some remnants may remain stable, and others may grow during follow-up [32]. Therefore, retreatment is necessary for patients with remnant necks with evidence of regrowth. We do not recommend observation of these documented cases because rebleeding can be an aggressive event causing permanent disability or death. Discriminating between complete and presumed complete clipping of aneurysms is challenging. To enhance procedural security, intraoperative video angiography with indocyanine or fluorescein, guided by a KINEVO 900 microscope, was used to confirm complete clipping. Postoperative follow-up with angiotomography at three and six months is crucial. For a better prognosis, postoperative DSA is recommended for ruptured aneurysms, even in the absence of neurological deficits.

## Conclusions

Recurrent MCA aneurysms after microsurgical clipping are rare but clinically significant, particularly when they exhibit aggressive regrowth and early rebleeding. This series highlights the multiple factors contributing to such recurrences, including residual neck remnants, the inherent fragility of the aneurysmal wall, and hemodynamic stress influenced by the type and positioning of surgical clips. Using the Kobayashi and Spiotta classification systems has been crucial in identifying recurrence patterns and mechanisms, thus helping to develop targeted and effective surgical interventions. Careful intraoperative techniques and advanced imaging methods such as intraoperative and postoperative DSA and CTA are essential for detecting subtle remnants and reducing the risk of recurrence. These imaging tools allow for precise assessments and adjustments during and after the procedure, which are vital for achieving optimal outcomes. This study emphasizes the urgent need for improved imaging protocols and optimized surgical strategies to tackle the challenges associated with early aneurysm regrowth. Additionally, it stresses the importance of conducting further research into the biological processes that drive these aggressive recurrences, including inflammation, endothelial dysfunction, and alterations in hemodynamics.
